# Effect of pretreatment with *Lactobacillus gasseri* OLL2716 on first-line *Helicobacter pylori* eradication therapy

**DOI:** 10.1111/j.1440-1746.2011.06985.x

**Published:** 2012-05

**Authors:** Ryuzo Deguchi, Hidemasa Nakaminami, Emiko Rimbara, Norihisa Noguchi, Masanori Sasatsu, Takayoshi Suzuki, Masashi Matsushima, Jun Koike, Muneki Igarashi, Hideki Ozawa, Ryuki Fukuda, Atsushi Takagi

**Affiliations:** * Gastroenterology, Tokai University School of MedicineIsehara, Kanagawa, Japan; † General Internal Medicine, Tokai University School of MedicineIsehara, Kanagawa, Japan; ‡ Department of Microbiology, School of Pharmacy, Tokyo University of Pharmacy and Life ScienceHorinouchi, Hachioji, Tokyo, Japan

**Keywords:** clarithromycin-resistant strain, *Helicobacter pylori*, *Helicobacter pylori* eradication therapy, *Lactobacillus gasseri*, probiotics

## Abstract

**Background and Aim:**

*Helicobacter pylori* eradication clearly decreases peptic ulcer recurrence rates. *H. pylori* eradication is achieved in 70–90% of cases, but treatment failures due to poor patient compliance and resistant organisms do occur. *Lactobacillus gasseri* can suppress both clarithromycin-susceptible and -resistant strains of *H. pylori in vitro*. The aim of this study was to determine the effect of pretreatment with *L. gasseri*- containing yogurt on *H. pylori* eradication. We conducted a randomized, controlled clinical trial in patients with *H. pylori* infection.

**Methods:**

A total of 229 patients were randomized into either a 1-week triple therapy of rabeprazole (10 mg bid), amoxicillin (750 mg bid), and clarithromycin (200 mg bid) or triple therapy plus *L. gasseri*-containing yogurt. In the yogurt-plus-triple therapy groups, yogurt containing *L. gasseri* OLL2716 (112 g) was given twice daily for 4 weeks (3 weeks pretreatment and also 1 week during eradication therapy). Clarithromycin resistance was determined by the detection of a mutation in 23S rRNA using nested polymerase chain reaction and the direct sequencing of DNA from pretreatment feces. *H. pylori* eradication was diagnosed based on the urea breath test and a stool antigen test after 8 weeks of eradication.

**Results:**

The status of *H. pylori* susceptibility to clarithromycin was successively determined in 188 out of 229 samples. The rate of infection with clarithromycin-resistant strains of *H. pylori* was 27.1%. Overall eradication (intention to treat/per protocol) was 69.3/74.5% for the triple-only group, and 82.6/85.6% for the yogurt-plus-triple group (*P* = 0.018/*P* = 0.041). Eradication of primary clarithromycin-resistant strains tended to be higher for yogurt-plus-triple therapy than triple-only therapy (38.5 *vs* 28.0%, respectively, *P* = 0.458).

**Conclusion:**

This study confirmed that the major cause of treatment failure is resistance to clarithromycin. A 4-week treatment with *L. gasseri*-containing yogurt improves the efficacy of triple therapy in patients with *H. pylori* infection.

## Introduction

*Helicobacter pylori* is a Gram-negative bacillus isolated from the gastric mucosa of patients with chronic gastritis.^1^ In Japan, approximately 50 million people are estimated to have *H. pylori* infection, and the infection rate in individuals aged ≥ 50 years is more than 70%.^2^*H. pylori* is also detected at a high rate in patients with gastric and duodenal ulcers. *H. pylori* eradication clearly prevents ulcer recurrence, and eradication therapy is now standard in the prevention of peptic ulcer recurrence.^3^*H. pylori*-positive chronic gastritis is often asymptomatic and has been associated with gastric malignancies, including gastric cancer^4^ and mucosa-associated lymphoid tissue (MALT) lymphoma. The annual incidence of gastric cancer in *H. pylori*-infected patients is about 0.5%, yet adequate treatment of chronic gastritis remains a major problem. First-line treatment for *H. pylori* eradication typically includes three drugs: a proton pump inhibitor (PPI) and the antibiotics amoxicillin and clarithromycin (CAM). Eradication clearly decreases peptic ulcer recurrence rates.^3^*H. pylori* eradication is achieved in 70–90% of cases, but treatment failures due to poor patient compliance and resistant organisms do occur. One reason for decreased patient compliance is diarrhea, often associated with antibiotic therapy. In addition, CAM, a first-line drug for community-acquired pneumonia, is widely prescribed for respiratory and oropharyngeal infections, thus increasing drug-resistant organisms. A surveillance study by the Japanese Society for *Helicobacter* Research reported that approximately 30% of *H. pylori* infections in Japan are resistant to treatment with CAM.

Probiotics are living microorganisms that improve the intestinal environment and inhibit harmful bacteria. *Lactobacilli* have been shown to exhibit beneficial effects on the stomach and inhibit *H. pylori*. *H. pylori* is able to colonize germ-free mice, but colonization does not occur in specific pathogen free (SPF) mice.^5^ In the mouse stomach, *Lactobacillus sp.* has demonstrated antimicrobial effects against *H. pylori in vitro* and in mouse models of *H. pylori* infection.^5^,^6^ We previously reported that yogurt-containing *Lactobacillus gasseri* (OLL2716) had a suppressive effect on *H. pylori* infection.^7^ The beneficial effects of fermented milk containing *Lactobacillus sp.* on *H. pylori* infection have also been reported.^8^,^9^ However, the administration of probiotics alone does not eradicate *H. pylori*. Recent evidence suggests that supplementation with probiotics could be effective in increasing eradication rates of anti-*H. pylori* therapy.^10^*L. gasseri* OLL2716 can suppress both CAM-susceptible and -resistant strains of *H. pylori in vitro* and in an *H. pylori*-infected murine model.^11^In order to determine the effect of pretreatment with *L. gasseri* contained in yogurt on *H. pylori* eradication, we conducted a randomized, controlled clinical trial in patients with *H. pylori* infection.

## Methods

### Patients

A total of 229 patients diagnosed with an *H. pylori* infection participated in this study from April 2008 to August 2010. Patients were defined as positive for *H. pylori* infection if the culture was positive, or if histology and rapid urease test were positive. The following exclusion criteria were applied: age below 18 or above 80 years, previous *H. pylori* eradication, and the use of antimicrobials or gastrointestinal medications like PPI within the previous 2 months. The ethics committee of Tokai University Hospital approved the protocol, and written informed consent was obtained from all patients.

### Determination of CAM resistance

CAM resistance was determined by the detection of a mutation in 23S rRNA using nested polymerase chain reaction (PCR) and direct sequencing of DNA from pretreatment feces.^12^ Briefly, pretreatment feces were pooled and kept in −20°C. DNA was extracted from feces using a bead-crushing method. Approximately 50 mg of feces was added to a tube with sodium phosphate buffer and 7.5 M guanidine solution and homogenized. The solution was centrifuged and subject to nested PCR. Nested PCR for the detection of mutations in the *H. pylori* 23S rRNA gene was performed. PCR primer pairs are shown in Table 1. DNA sequencing was carried out using an ABI PRISM 3100 DNA sequencer (Applied Biosystems, Carlsbad, CA, USA).

**Table 1:** Oligonucleotide primers used for nested-PCR

PCR	Primer name	Sequence (5′ to 3′)	Tm (°C)	Size of PCR product (bp)	Primer position in 23SrRNA†
1st PCR	F	GGTCTCAGCAAAGAGTCCCT	62.4	493	1835
	R	CCCACCAAGCATTGTCCT	63.6		2327
2nd PCR	F-nested	AGGATGCGTCAGTCGCAAGAT	68.2	367	1942
	R-nested	CCTGTGGATAACACAGGCCAGT	67.1		2308

† Position in 23S rRNA gene of *H. pylori* strain 26695 (GenBank accession no. AE000569) was shown.

F, forward primer for 1st PCR; F-nested, forward primer for 2nd PCR; PCR, polymerase chain reaction; R, reverse primer for 1^st^ PCR; R-nested, reverse primer for 2^nd^ PCR.

### Yogurt

The yogurt containing *L. gasseri* OLL2716 (≥ 10^9^c.f.u.) was obtained from Meiji Dairies Corporation, Tokyo, Japan.

### Protocol

A total of 229 patients were randomized either to 1 week of triple therapy comprising rabeprazole (10 mg bid), amoxicillin (750 mg bid), and clarithromycin (200 mg bid) or triple therapy plus *L. gasseri*-containing yogurt. In the yogurt-plus-triple therapy group, yogurt containing *L. gasseri* OLL2716 (112 g) was consumed twice daily for 4 weeks (3 weeks pretreatment followed by 1 week during eradication therapy). The triple therapy regimen in this study used the same doses based on a large-scale study in Japan.^13^ Randomization was carried out according to a computer-generated randomization list. As a combination of the urea breath test (UBT) and a stool antigen test is useful for the clinical evaluation of eradication therapy,^14^*H. pylori* eradication was diagnosed based on UBT and stool antigen test after 8 weeks of eradication. For discordant results between UBT and stool antigen test, endoscopic biopsy specimens were obtained and *H. pylori* culture was carried out.

### ^13^C-urea breath test

The urea breath test was conducted 8 weeks after the end of eradication therapy. It was performed after overnight fasting using 100 mg ^13^C-urea tablets (Dainippon Sumitomo Pharmaceutical Co., Tokyo, Japan). Breath samples were collected before and 20 min after the ingestion of ^13^C-urea. The cut-off value of ^13^C value was defined as 3.5‰.^15^

### *H. pylori* stool antigen test

Stool samples were frozen at −70°C until assaying. The investigators were blinded to the results of other *H. pylori* tests. A commercial EIA (Testmate *H. pylori*; Wakamoto Pharmaceutical, Tokyo, Japan) was used to detect *H. pylori*, according to the manufacturer's instructions. About a 100-mg fecal sample was diluted in 0.4 mL of dilution buffer. Fifty microliters of diluted fecal sample and peroxidase-conjugated monoclonal antibody were added to each well of microtiter plates, and the plates were incubated for 1 h at 25°C. The absorbance at dual wavelengths (450 and 630 nm) was measured on a microplate reader. The cut-off value for the stool antigen test was < 0.100 for a negative and ≥ 0.100 for a positive result.^15^

### Statistical analysis

**Figure 1:**
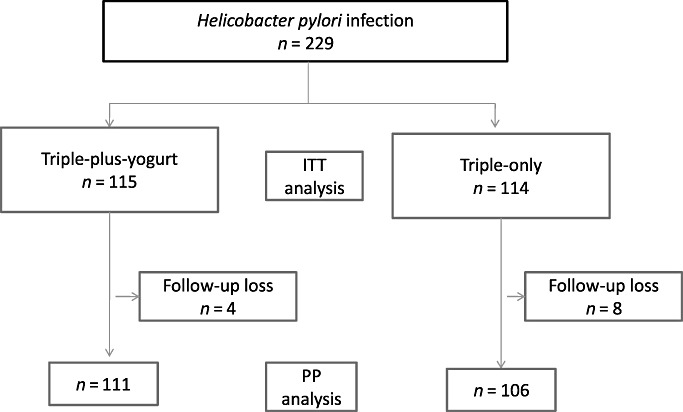
Flow schematic of the study involving intention-to-treat (ITT) and per-protocol (PP) analyses.

The number of patients required for the study was calculated such that a difference of 17% in the eradication rates between the two study groups could be detected. Thus we calculated that at least 110 patients per group were required to give the study 80% power at a significant level of 5%. Eradication rates were determined for both groups employing per-protocol (PP) and intention-to-treat (ITT) analyses. For ITT analysis, all enrolled patients were included. For PP analysis, those who had not undergone the UBT and stool antigen test, or those who had taken less than 70% of any drugs, were excluded (Fig. 1).

Eradication rates were compared using the χ^2^-test. Significance was set at *P* < 0.05. Statistical analysis was performed using spss 17.0J Windows (spss Japan, Inc., Tokyo, Japan).

## Results

### Demographic background

Demographic characteristics and adverse effects are shown in Table 2. There were no significant differences in demographic features and endoscopic diagnoses. *H. pylori* was diagnosed by either the UBT or *H. pylori* culture during endoscopic examination. There were no significant differences between the two groups regarding adverse effects.

**Table 2:** Demographic characteristics of the two groups

Variables	Yogurt-plus-triple group	Triple-therapy-only group	*P*-values
	*n* = 115	*n* = 114	
Mean age (years)	55.9	57.8	0.75
Women	39 (33.9%)	48 (42.1%)	0.202
PUD	87 (75.7%)	82 (71.9%)	0.552
Clarithromycin resistance	26/96 (27.1%)	25/92 (27.2%)	0.981
Side-effect			
Diarrhea	6	4	0.748

PUD, peptic ulcer disease.

### *H. pylori* susceptibility to CAM

The status of *H. pylori* susceptibility to CAM was successively determined in 188 of 229 samples. DNA sequencing of the *H. pylori* 23S rRNA gene without mutation (wild type) was archived in 137 patients. The 23S rRNA gene with 2142G or A2143G mutation was detected in 51 patients. Mixed infections with both CAM-susceptible and-resistant *H. pylori* were detected in 13 samples.

### *H. pylori* eradication rates

UBT and stool antigen test results were consistent except in one patient. This discordant case was followed by endoscopic examination, and was revealed to be a false-positive result of the stool antigen test.

Based on ITT analysis, eradication rates in the yogurt-plus-triple and triple-only groups were 82.6 and 69.3%, respectively (*P* = 0.018) (Table 3). PP analysis also showed that *H. pylori* eradication rates in the yogurt-plus-triple group (85.6%) were significantly higher than those in the triple-therapy-only group (74.5%) (*P* = 0.041). Eradication rates of CAM-sensitive *H. pylori* were high in both groups (Table 4). Eradication of primary CAM-resistant strains tended to be higher in the yogurt-plus-triple group than in the triple-therapy-only group (38.5 *vs* 28.0%, respectively, *P* = 0.428). In addition, in mixed infections with both CAM-susceptible and -resistant *H. pylori*, the eradication rate in the yogurt-plus-triple group was 50% (3/6), however, that in the triple-therapy-only group was 0% (0/4). One patient in the yogurt-plus-triple group and two patients in the triple-therapy-only group were lost to follow up.

**Table 3:** *Helicobacter pylori* eradication rate

	Yogurt-plus-triple group	Triple-therapy-only group	*P*-value
ITT analysis			
Eradication rate	82.6% (95 of 115)	69.3% (79 of 114)	0.018
95%CI	75.7–89.5%	60.8–78.3%	
PP analysis			
Eradication rate	85.6% (95 of 111)	74.5% (79 of 106)	0.041
95%CI	78.9–92.1%	66.2–82.8%	

CI, confidence interval; ITT, intention-to-treat; PP, per-protocol.

**Table 4:** *H. pylori* eradication rates in CAM-susceptible and -resistant infection

	Yogurt-plus-triple group	Triple-therapy-only group	*P*-value
CAM-susceptible			
ITT analysis	*n* = 70	*n* = 67	
Eradication rates	92.8% (65 of 70)	85.6% (58 of 67)	0.224
95%CI	86.8–98.8%	77.2–94.0%	
PP analysis	*n* = 56	*n* = 54	
Eradication rates	95.6% (65 of 68)	93.5% (58 of 62)	0.607
95%CI	90.7–100%	87.5–99.5%	
CAM-resistant			
ITT analysis	*n* = 26	*n* = 25	
Eradication rates	38.5% (10 of 26)	28.0% (7 of 25)	0.428
95%CI	19.8–57.2%	10.4–45.6%	
PP analysis	*n* = 25	*n* = 23	
Eradication rates	40% (10 of 25)	30.4% (7 of 23)	0.489
95%CI	20.8–59.2%	11.7–49.1%	

CAM, clarithromycin; CI, confidence interval; ITT, intention-to-treat; PP, per-protocol.

## Discussion

In the present study, 4-week pretreatment with yogurt containing *L. gasseri* before triple therapy improved the eradication rates. The use of probiotics for *H. pylori* infection was first adopted following a series of research studies in germ-free mice. The studies reported that *H. pylori* colonizes germ-free but not SPF mice, and that *Lactobacillus* in the stomach of SPF mice inhibits colonization by *H. pylori*.^5^,^6^ In a subsequent study involving human volunteers, *L. gasseri* (OLL2716) decreased the *H. pylori* density and improved gastritis.^7^ Several studies also reported that the ingestion of fermented milk containing *Lactobacillus* improved *H. pylori*-infected gastritis, but eradication was not successful, and, after stopping ingestion, this effect on *H. pylori* suppression was lost. Recent evidence revealed that supplementation with probiotics could be effective in increasing eradication rates due to anti-*H. pylori* therapy. Tong *et al*.^10^conducted a meta-analysis of supplemental probiotics in eradication therapy. Among 14 randomized trials, the eradication rates for eradication therapy alone and eradication therapy with probiotics were 74.8 and 83.6%, respectively. With combined treatment, the eradication rate increased, and adverse effects, such as diarrhea, decreased. However, the eradication rate varies by protocol. Typically, probiotics were given during the eradication therapy or following 3–4 weeks.^16^–^18^ In contrast, Sheu *et al*.^19^ reported that pretreatment with *Lactobacillius* and *Bifidobacterium*-containing yogurt improved the efficacy of quadruple therapy after failed triple therapy. They also demonstrated a decreased bacterial load after pretreatment with yogurt. Therefore, we chose a protocol involving pretreatment with *L. gasseri*-containing yogurt. Based on ITT analysis, eradication rates in the yogurt-plus-triple and triple-therapy-only groups were 82.6% and 69.3%, respectively (*P* = 0.018) (Table 3). PP analysis also showed that *H. pylori* eradication rates in the yogurt-plus-triple group (85.6%) were significantly higher than those in the triple-therapy-only group (74.5%) (*P* = 0.046).

The mechanism by which probiotics reduce *H. pylori*-related gastric mucosal injury has not been elucidated. Gastric mucosal inflammation due to *H. pylori* infection is primarily mediated by cytokines. Cytokines involved in the clinical manifestations of *H. pylori* infection of the gastric epithelium include interleukin (IL)-1, IL-8, and tumor necrosis factor.^20^,^21^ In *H. pylori* infection, neutrophil infiltration is a characteristic histologic finding. IL-8 is a neutrophil chemotactic factor produced by *H. pylori*-infected gastric epithelium. Previously, we reported IL-8 concentrations in the gastric mucosa measured before and after *L. gasseri*-containing yogurt consumption. *L. gasseri* consumption significantly decreased IL-8, whereas with a placebo, there was no decrease in IL-8.^22^ In that 8-week study, although eradication was not observed, some volunteers showed histologic improvement of gastritis.

The major cause of eradication failure was CAM-resistance due to mutation of the 23S rRNA gene. Eradication rates of CAM-susceptible *H. pylori* were high in both groups (Table 4). Eradication rates of primary CAM-resistant strains tended to be higher in the yogurt-plus-triple group than that in triple-only group (38.5 *vs* 28.0%, respectively, *P* = 0.428). The detection of CAM-resistance based on analyzing feces is a non-invasive method. Furthermore, mixed infections with both CAM-susceptible and -resistant *H. pylori* were detected in 13 patients.

In mixed infections with both CAM-susceptible and -resistant *H. pylori*, the eradication rate in the yogurt-plus-triple group was 50% (3/6), however, that in the triple-therapy-only group was 0% (0/4).

In conclusion, our data suggested that supplementation with yogurt containing *L. gasseri* is effective for first-line eradication therapy. A further large-scale study is required to clarify the effectiveness of *L. gasseri* against CAM-resistant *H. pylori* infection.
